# *Phellinus linteus* sensitises apoptosis induced by doxorubicin in prostate cancer

**DOI:** 10.1038/sj.bjc.6603277

**Published:** 2006-07-25

**Authors:** L Collins, T Zhu, J Guo, Z J Xiao, C-Y Chen

**Affiliations:** 1 Department of Pathology, K522, Boston University School of Medicine, 80 East Concord Street, Boston, MA 02118, USA; 2 Department of Biochemistry, Boston University School of Medicine, 80 East Concord Street, Boston, MA 02118, USA

**Keywords:** *Phellinus linteus*, doxorubicin, apoptosis, caspase, prostate cancer

## Abstract

It has been demonstrated that the *Phellinus linteus* (PL) mushroom, which mainly consists of polysaccharides, possesses antitumour activity. The mechanisms of PL against malignant growth remain unknown. The anticancer drug doxorubicin (Dox) has been shown to induce apoptosis via initiating a caspase cascade. In this investigation, we tested the effect of PL on Dox-induced apoptosis in prostate cancer LNCaP cells. We showed that PL or Dox, at relatively low doses, does not induce apoptosis in the cells. However, combination treatment with low doses of PL and Dox results in a synergistic effect on the induction of apoptosis. In this apoptotic process, caspases 8, 3 and BID are cleaved, and the addition of caspase inhibitor z-VADfmk completely blocks apoptosis. In addition, JNK is activated in response to PL or the combination treatment in LNCaP cells. The suppression of JNK partially inhibits the induction of apoptosis elicited by the co-treatment. These findings indicate that PL has a synergistic effect with Dox to activate caspases in prostate cancer LNCaP cells. Our study also suggests that PL has therapeutic potential to augment the magnitude of apoptosis induced by antiprostate cancer drugs.

A major challenge in the treatment of prostate cancer, especially the hormonally refractory form, is the presence of genetic and cellular abnormalities associated with tumorigenesis. Therefore, development of less toxic, more effective treatments is necessary. One strategy is to explore and understand the mechanisms of action of natural medicines. Fungi, like yeast, algae, bacteria and higher plants have been intensively investigated for antitumour modality, because it appears that they are able to modulate the body's immune responses against tumours with very low toxic potential (Oh *et al*, 1992; Song *et al*, 1995; Borchers *et al*, 1999; Han *et al*, 1999). Studies indicate that the main components in these basidiomycetes are polysaccharides (Lorenzen and Anke, 1998; Reshetnikov *et al*, 2001; Wasser, 2002). Beta-glucocans isolated from lentinan (*Lentinus edodes*), pachymaran (*Poria cocos*), schizophyllan (*Schizophyllum commune*) and krestin (*Coriolus versicolor*) have been demonstrated to stimulate lymphocytes and elicit nonspecific immune-activities in various experimental settings (Wasser, 2002). It has also been reported that *Phellinus linteus* (PL) has the most potent effect among basidiomycetes in antitumour action (Wasser, 2002). Studies have also shown that PL extraction is able to strongly suppress the growth of various tumours *in vitro* and *in vivo* (Chihara *et al*, 1969; Chung *et al*, 1982; Cun *et al*, 1994), including the induction of growth arrest or apoptosis. However, the molecular signalling involved in PL-mediated antitumour activity has not yet been fully explored.

Caspases are key apoptotic effectors and belong to a family of cysteine-containing, aspartate-specific proteases (Alnemri *et al*, 1996; Nicholson and Thomberry, 1997). Caspases exist as dormant proenzymes in cells and are activated through proteolysis. Once activated, caspases cleave a host of cellular substrates, leading to morphological hallmarks of apoptosis, including DNA fragmentation. In a caspase cascade, caspase 8 or 9 acts as the initiator, whereas others (e.g., caspases 3 and 7) serve as effectors of apoptosis (Cohen, 1997; Salvesen and Dixit, 1997). Several anticancer drugs, such as doxorubicin (Dox), have been suggested to elicit a caspase cascade, leading to apoptosis (Hannun, 1997; Los *et al*, 1997). A low dose of Dox can activate the G_1_ checkpoint via a p53-dependent pathway, and high doses of Dox cause apoptosis (Hannun, 1997; Los *et al*, 1997). Studies have also shown that Dox can synergise with other drugs to induce cytotoxic effects against breast cancer, hepatocellular carcinoma and colon carcinoma (Keane *et al*, 1999; Muzutani *et al*, 1999; Yamanaka *et al*, 2000; Lacour *et al*, 2001). The combination treatment of Dox and TRAIL dramatically augments the therapeutic effect against prostate cancer (Wu *et al*, 2002).

JNK, a c-Jun NH_2_-terminal kinase, was discovered to bind to the c-Jun transactivation domain and phosphorylate Ser-63, Ser-73 and some secondary sites (Devary *et al*, 1991; Hibi *et al*, 1993). JNK is rapidly phosphorylated and subsequently activated in response to various stimuli, including different types of stresses (Devary *et al*, 1991; Hibi *et al*, 1993). Studies have shown that JNK can promote apoptosis by regulating BID processing or c-FLIP_L_ degradation (Derijard *et al*, 1994; Davis, 2000). Upon activation, JNK phosphorylates Itch (a E3-related ligase) and accelerates the decay of c-FLIP_L_, which augments caspase 8 activity and allows apoptosis to proceed (Gao *et al*, 2004; Chang *et al*, 2006).

Based on the information that Dox can synergise with TRAIL to elicit cytotoxicity in various types of tumours (Hannun, 1997; Los *et al*, 1997; Keane *et al*, 1999; Muzutani *et al*, 1999; Yamanaka *et al*, 2000; Lacour *et al*, 2001; Wu *et al*, 2002) as well as that PL is able to interfere with tumour growth, we tried to explore the potential of the combination treatment of Dox and PL against prostate cancer. We examined LNCaP prostate cancer cells looking for evidence of synergy between Dox and PL, and specifically to identify the molecular mechanisms underlying this synergistic effect. In the cells tested, PL enhanced Dox-induced cell death, possibly via caspase-mediated apoptotic signalling pathways. During this process, caspases 8, 3 and BID were activated by the combination treatment of low doses of PL and Dox. The apoptotic process could be blocked by the addition of caspase inhibitor z-VADfmk. *Phellinus linteus* itself could also upregulate JNK activity and further reduce the expression level of c-FLIP_L_. In addition, the suppression of JNK partially blocked apoptosis induced by the combination treatment of PL and Dox. The data suggest that PL may act through interfering with antiapoptotic factor c-FLIP_L_ to sensitise Dox-mediated apoptotic signalling. Our study also implies that the combination of PL and Dox may have potential for the development of a more effective treatment against prostate cancer.

## MATERIALS AND METHODS

### Cell culture and treatments

The human prostate cancer LNCaP cells were purchased from American Tissue Culture Collection (Rockville, MD, USA) and cultured in Dulbecco's modified Eagle's medium (DMEM) supplemented with 10% heat-inactivated fetal calf serum (FCS) 2 mM L-glutamine, 100 U ml^−1^ of penicillin, 100 g ml^−1^ of streptomycin. Normal human prostate epithelial PrEC cells (Cambrex, One Meadowlands Plaza, East Rutherford, NJ, USA) were cultured in the PrEGM medium (Cambrex, NJ, USA). Whole powdered PL that had been grown on germinated brown rice was purchased from Panbio-Tech (Taejon, Korea), and purified using ethanol precipitation methods, followed by DEAE-cellulose and gel permeation chromatography (Song *et al*, 1995). The purified components of PL mostly consist of polysacharides. Doxorubicin was purchased from Sigma, St Louis, MO, USA.

### Flow cytometric determination of nuclear DNA fragmentation assay

A flow cytometric analysis was performed with a FACScan (Becton-Dickenson, Mountain View, CA, USA). The data analysis and display were performed using the Cell-Fit software program (Becton-Dickenson). Cell-Fit interprets data from the flow cytometer and provides real-time statistical analysis of the data, computed at 1 s intervals and also discriminates doublets or adjacent particles. Briefly, following various treatments, cells (1 × 10^6^ ml^−1^) were washed with 1 × phosphate–buffered saline (PBS), fixed with 70% ethanol, and treated with 10 ng ml^−1^ RNAse. Subsequently, cells were stained with propidium iodide (5 *μ*g ml^−1^). The stained samples were kept in the dark at 4°C overnight before flow cytometric analysis.

### [^3^H]Thymidine incorporation assay

After treatment, cells growing in 24-well plates were starved for 48 h. The growth medium containing [^3^H]thymidine (2 *μ*Ci ml^−1^) was then added, with or without treatment. Twenty-four hours later, the medium was aspirated and cells were fixed. After washing with 1 ml of 5% trichloroacetic acid, cells were solubilised in 1% sodium dodecyl sulphate (SDS) and 0.3 nM NaOH. Incorporated radioactivity was determined by scintillation counter.

### Immunoblot

After treatment, cells were washed in 1 × PBS and then lysed in the lysis buffer (50 mM Tris-HCl, pH 8.0, 150 mM NaCl, 1% Triton X-114, 0.5% sodium deoxycholate, 0.1% SDS, containing 1 mM phenylmethylsulphonyl fluoride, 1 *μ*g ml^−1^ aprotinin, 1 *μ*g ml^−1^ leupeptin, 1 *μ*g ml^−1^ pepstatin A) on ice for 30 min. The total protein concentrations in the cell lysates were normalised and adjusted to 0.4 M NaCl, 0.5% deoxycholate and 0.05% SDS for immunoblotting (Denis *et al*, 2003). The samples were separated on a 10% SDS–polyacrylamide gel electrophoresis gel and subsequently transferred to a nitrocellulose membrane. All the antibodies used were purchased from BD Biosciences (San Diego, CA, USA).

### Caspase activity assay

After various treatments, lysates were prepared and the activities of caspases 3 and 8 were measured by a colorimetric analysis (Wu *et al*, 2001).

### Preparation of subcellular fractions

After the treatments, cells (1 × 10^7^) were washed twice with 1 × PBS and resuspended in 1 ml of 1% Triton X-114 lysis buffer (Denis *et al*, 2003). The cell suspensions were transferred to a 1-ml syringe and sheared by being passed 40 times through a 25-G needle. The lysates were centrifuged at 280 g for 10 min, and the supernatant was centrifuged at 16 000 *g* for 30 min. Afterwards, the supernatant was collected as the cytosolic fraction. For the mitochondrial fraction, the cells (1 × 10^9^) were resuspended in buffer A (50 mM Tris, pH 7.5, 1 mM ethyleneglycol tetraacetate, 5 mM 2-mercaptoethanol, 0.2% bovine serum albumin, 10 mM KH_2_PO_4_, pH 7.6, 0.4 M sucrose), and allowed to swell on ice for 40 min. After centrifugation, the resulting pellets were resuspended in buffer B (10 mM KH_2_PO_4_, pH 7.2, 0.3 mM mannitol, 0.1% bovine serum albumin). The mitochondrial fractions were subsequently separated on a sucrose step gradient (Denis *et al*, 2003).

### Soft agar assay

Petri dishes were first layered with 0.6% basal agar dissolved in DMEM medium containing 10% FCS. Cells were mixed in 0.33% agar dissolved in DMEM medium containing PL (1 mg ml^−1^). The plates were incubated at 37°C in a humidified atmosphere for 14 days. Fresh medium (2 ml) containing PL (1 mg ml^−1^) was added to the cultures every 3 days.

## RESULTS

### PL mushroom and doxorubicin, at a low dose, synergise to induce apoptosis in prostate cancer cells

**Figure 1:**
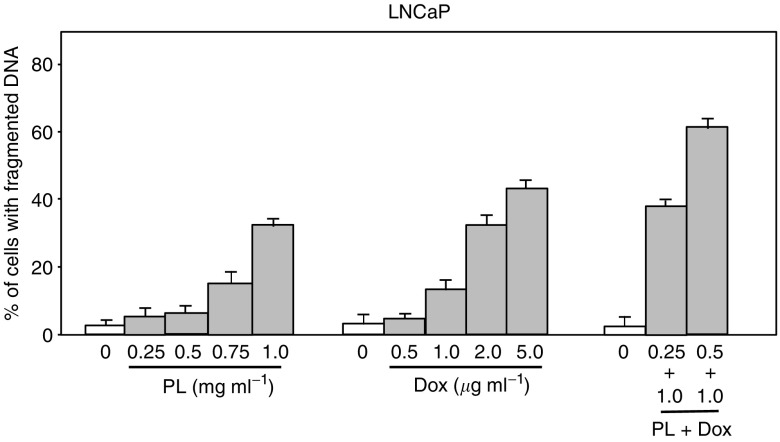
DNA fragmentation and [^3^H]thymidine incorporation in response to the treatment of PL, Dox and PL plus Dox. (**A**) and (**B**) LNCaP or PrEC cells were cultured in the growth medium in the presence of different concentrations of PL, Dox, or PL plus Dox. After treatment for 48 h, the percentage of cells with fragmented DNA was analysed by a flow cytometer. Error bars represent the s.d. over five independent experiments. (**C**) Following serum-starvation (0.5% serum) for 48 h, the cells were refed with growth medium containing 10% serum and [^3^H]thymidine (2 *μ*Ci ml^−1^) in the presence or absence of PL, Dox or both for 24 h. Subsequently, trichloroacetic acid-insoluble radioactivity was determined. Error bars represent the s.d. from five independent experiments.

It is known that PL can interfere with tumour proliferation, and the antitumour effect of PL has been indicated to be related to the contents of polysacharides (Chihara *et al*, 1969; Chung *et al*, 1982; Cun *et al*, 1994). However, the mechanisms of PL that suppress tumour growth remain unknown. To further investigate the antitumour effect of PL in prostate cancer, we tested the effect of the combination treatment of PL and Dox, at a low dose range, on human prostate cancer LNCaP cells that have been shown to be sensitive to Dox treatment (Hannun, 1997; Los *et al*, 1997; Keane *et al*, 1999; Muzutani *et al*, 1999; Yamanaka *et al*, 2000; Lacour *et al*, 2001; Wu *et al*, 2002). LNCaP cells were either left untreated, or treated with different doses of PL, Dox or the combination of these two for 48 h. Afterwards, a DNA fragmentation assay was conducted (Figure 1A). Under normal growth conditions, only a few untreated LNCaP cells had fragmented DNA. After treatment with PL alone, the percentage of DNA fragmentation was moderately increased in a dose-dependent manner. A similar phenomenon was observed following Dox treatment; low doses of Dox had a minor apoptotic effect on the cells. In comparison, treatment with high doses of Dox (>2 *μ*g ml^−1^) for 24 h caused more than 40% of the cells to have fragmented DNA, also in a dose-dependent manner. As low doses of PL or Dox could not induce apoptosis, we tested whether the combination of these two would affect cell viability. The cells were treated with 0.25 or 0.5 mg ml^−1^ of PL plus 1 *μ*g ml^−1^ of Dox. Twenty-four hours after the combination treatment, the percentage of DNA fragmentation was analysed. A significant synergy of PL and Dox in the induction of apoptosis was observed. Normal prostate epithelial PrEC cells were also used for testing the synergistic effect on the induction of apoptosis (Figure 1B). Neither the drug alone nor the combination treatment elicited apoptosis in the normal cells. To further define the growth pattern of PrEC cells in response to the combination treatment, a [^3^H]thymidine incorporation assay was performed (Figure 1C). Serum-starved PrEC cells incorporated a large amount of [^3^H]thymidine into their genome after adding the growth medium containing 10% serum. In contrast, cells under the same growth conditions did not incorporate [^3^H]thymidine in response to the treatment of PL, Dox or both. Overall, the results indicate that PL, at low doses, can render the prostate cancer cells, but not the normal prostate epithelial cells, susceptible to Dox-induced apoptosis.

To further define the long-term effect of the combination treatment of PL and Dox on tumour growth, a soft agar assay was performed (Table 1). Untreated LNCaP cells formed colonies in soft agar medium. Interestingly, although low doses of PL or Dox did not affect the viability of the cells (see Figure 1), it moderately blocked the formation of the colonies. This may be due to the drugs’ negative effect on cell cycle progression (Hannun, 1997; Los *et al*, 1997), which inhibits the cells from forming colonies. The high dose of Dox treatment (5 *μ*g ml^−1^) completely prevented the cells from forming colonies. The combination treatment of the low doses of PL and Dox achieved a prominent blocking effect on colony formation. Thus, the data suggest the existence of a synergistic, cytotoxic effect of PL and Dox, at low doses, on prostate cancer cells.

### Involvement of caspases in the synergistic effect of PL and Dox

**Figure 2:**
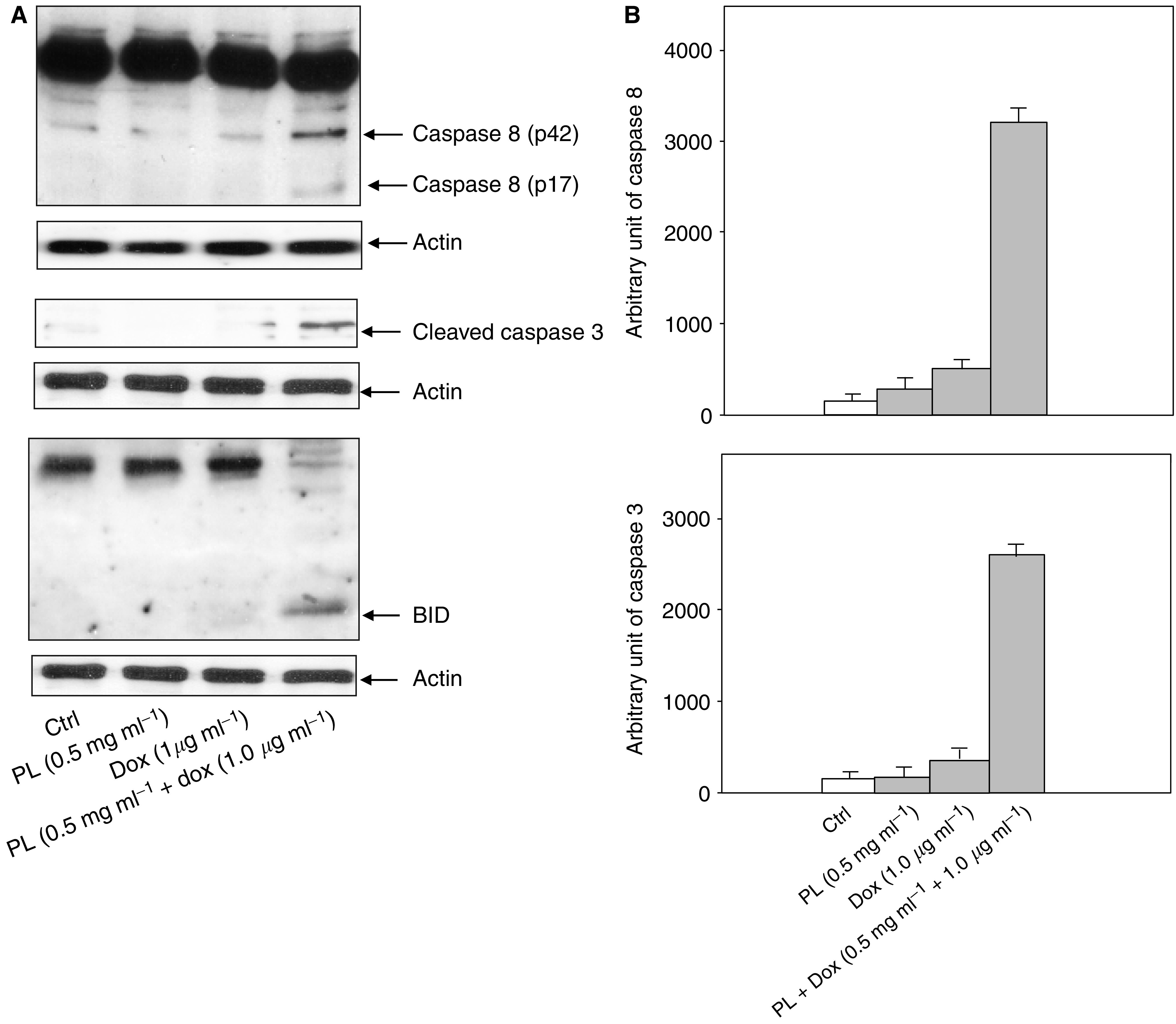
Activation of caspase 3, 8 or BID following the treatmet with PL, Dox or PL plus Dox. (**A**) After treating with PL, Dox, or PL plus Dox, LNCaP cells were harvested and lysates were prepared. The existence of activated forms of caspases 3, 8 and BID was determined by Western blot. Equal loading of total proteins was verified by *β*-Actin expression. (**B**) Following the treatments, lysates were prepared to measure the activity of these caspase family members using a colorimetric assay. Error bars represent the s.d. over five independent experiments.

The caspase cascade plays an important role in many types of apoptosis, including Dox-induced apoptosis (Hannun, 1997; Los *et al*, 1997). It is known that Dox, at high doses, can induce apoptosis, possibly through caspase-mediated apoptotic signalling, including caspase 8 (Hannun, 1997; Los *et al*, 1997). To determine whether and which caspases are involved in the synergistic induction of apoptosis by PL and Dox, our primary focus was to study the mechanisms of the combination treatment of PL and Dox on several caspase family members. Caspases, upon activation, are cleaved into a small, active form (Hannun, 1997; Los *et al*, 1997). Therefore, immunoblot analysis was employed using caspases 3, 8 and BID antibodies to detect the presence and levels of the corresponding proteins following various treatments (Figure 2A). The active, cleaved forms of caspases 3, 8 and BID were absent in LNCaP cells upon treatment with low doses of PL or Dox. In comparison, the caspases were significantly activated in response to the combination treatment. To further determine the activation of caspases by the co-treatment of PL and Dox, the activities of caspases 3 and 8 were measured using an enzymatic assay (Figure 2B). Again, the activities of these proteases were undetectable following treatment with low doses of PL or Dox alone. The combination treatment dramatically upregulated the activities of these caspases.

**Figure 3:**
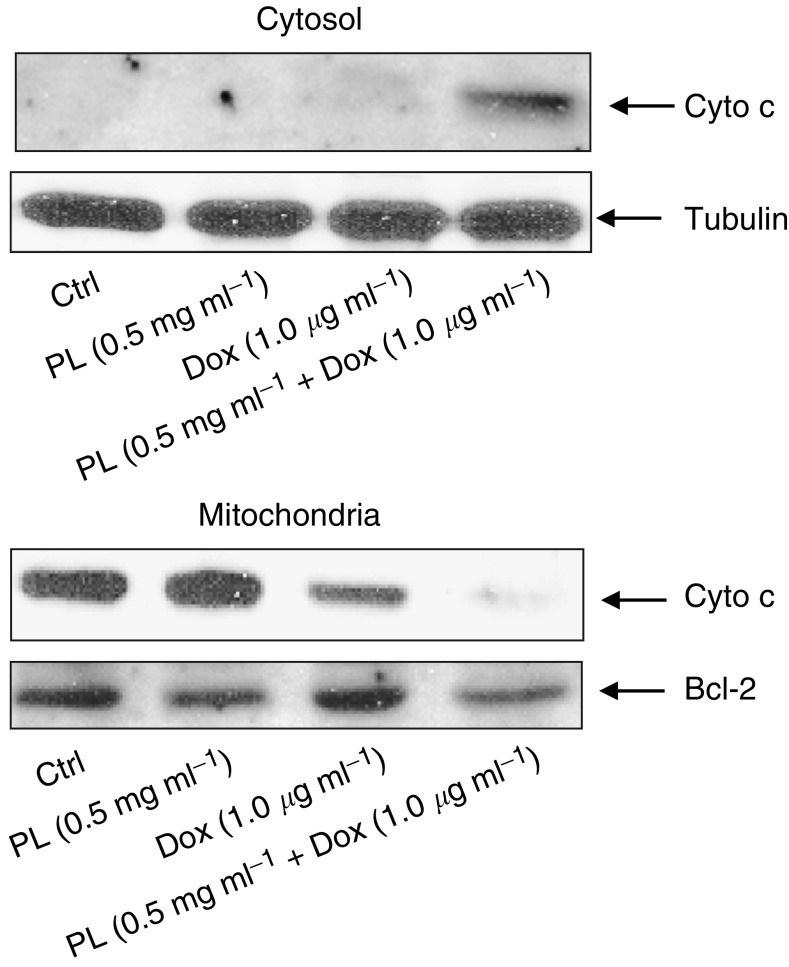
Releasing of cytochrome *c* to the cytosol following the treatment with PL, Dox, or PL plus Dox. The mitochondrial or cytosolic fractions from untreated or treated cells were isolated and analysed for the expression of cytochrome *c* by Western blot. Equal loading of proteins in the mitochondrial or cytosolic fraction was determined by reprobing the blot with antitubulin or Bcl-2 Ab.

Cytochrome *c* is an apoptotic executor during the process of caspase-mediated apoptosis. Therefore, the release of cytochrome *c* from the mitochondria to the cytosol in response to various treatments was also examined in LNCaP cells (Figure 3). After isolating the cytosolic or mitochondrial fractions from untreated or treated LNCaP cells, immunoblotting was performed using an anti-cytochrome *c* antibody. Cyctochrome *c* was present in the cytosolic fraction isolated from the cells treated with the combination of PL and Dox, but not in the cells either untreated or treated with PL or Dox alone (Figure 3, upper panel). Also, after the addition of low doses of PL plus Dox, the anti-cytochrome *c* antibody could not detect the protein in the mitochondrial fraction of the cells, but the protein was present in the mitochondrial fraction of either untreated cells or cells treated with PL or Dox (Figure 3, lower pannel). Overall, these results indicate that low doses of PL and Dox can synergise to enhance caspase activity in LNCaP cells.

### JNK is activated and controls FLIP_L_ expression in response to PL or PL plus Dox

**Figure 4:**
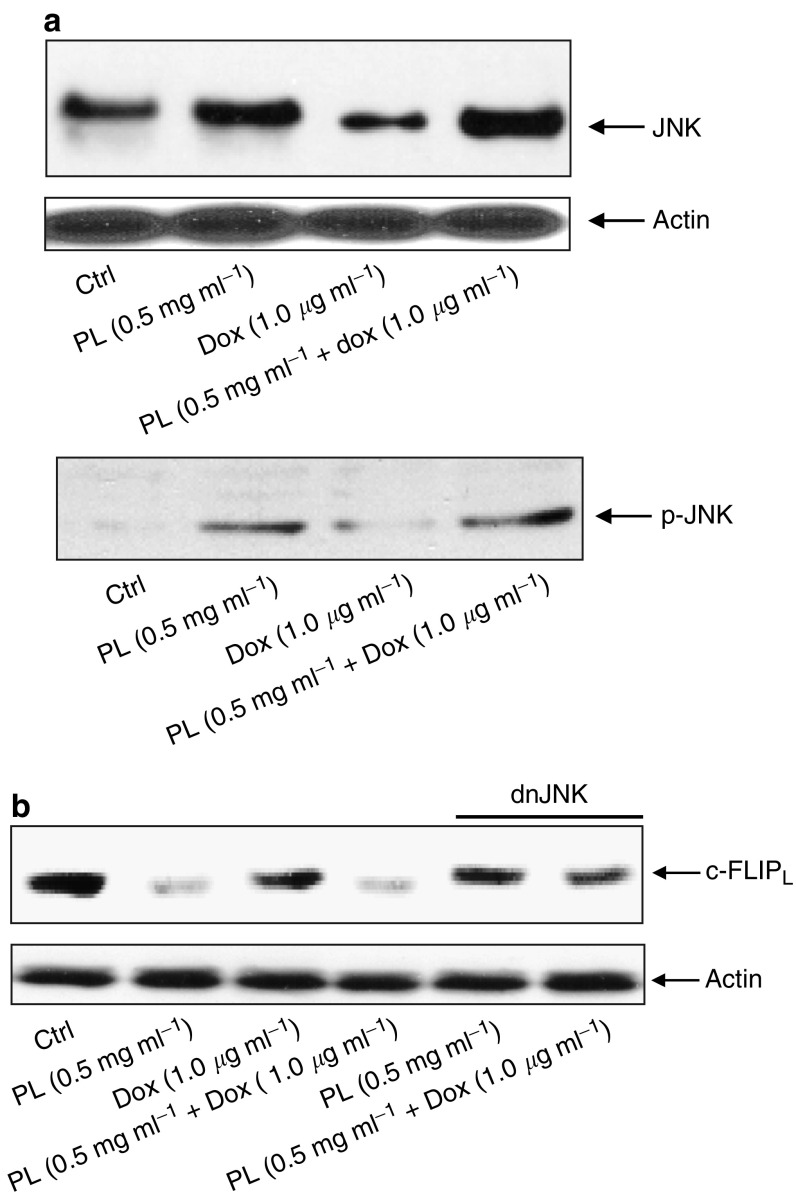
JNK activation and c-FLIP_L_ expression. (**A**) After treatment with PL, Dox or PL plus Dox, cells were harvested and lysates were prepared. The protein expression level of JNK or the presence of the phosphorylated form of JNK was determined by Western blot using the corresponding antibodies. Equal loading of total proteins in each sample was verified by *β*-actin. (**B**) c-FLIP_L_ expression upon treatment with PL, Dox, or PL plus Dox. The cells with or without addition of a *dn-JNK* were treated with PL, Dox, or PL plus Dox. Subsequently, lysates were prepared to analyse the expression level of c-FLIP_L_. The percentages of the cells with fragmented DNA were determined by flow cytometry. The error bars represent the s.d. over five independent experiments.

JNK, a stress-related kinase, requires phosphorylation to be activated and is often involved in the modulation of various types of apoptosis, including those induced by caspases (Davis, 2000). It has been shown that JNK activation in tumour necrosis factor (TNF)*α*-induced apoptotic processes promotes the activity of caspases by regulating the expression level of c-FLIP_L_, a caspase cascade inhibitor (Chang *et al*, 2006). To determine the JNK activation status in our experimental settings, the expression of JNK was analysed by immunoblotting using an anti-JNK antibody. JNK protein expression was detectable in untreated LNCaP cells or cells treated with Dox (Figure 4A, upper panel). In comparison, PL treatment alone or the combination treatment augmented the expression of this kinase. As JNK is phosphorylated upon activation, its phosphorylation status was then determined using the anti-phos-JNK antibody (Figure 4A, lower panel). The phosphorylated JNK was recognised by the antibody in the cells treated with PL alone or co-treated with PL plus Dox. JNK was not activated by the addition of Dox alone.

JNK has been demonstrated to regulate the degradation of c-FLIP_L_ that is an important negative regulator of caspase 8 and further controls the expression of the protein (Chang *et al*, 2006). We conducted immunoblotting experiments to test the expression of c-FLIP_L_ in LNCaP cells (Figure 4B). The amount of c-FLIP_L_ expression in the cells treated with Dox alone was the same as that in untreated cells. However, c-FLIP_L_ expression was dramatically suppressed by PL or the combination treatment. In addition, blockade of JNK expression by introduction of a dominant-negative *JNK* into the cells lifted the inhibitory effect on the expression of c-FLIP_L_ rendered by the treatment with PL or PL plus Dox. The data suggest that PL, via regulating JNK activation and subsequent c-FLIP_L_ expression, synergises with Dox for the initiation of a caspase cascade.

**Figure 5:**
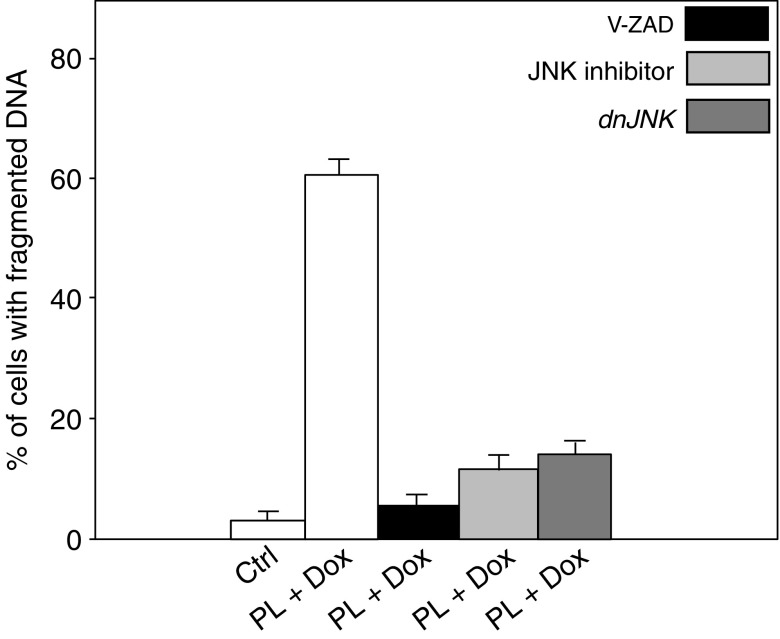
Effect of JNK inhibition on apoptosis induced by the combination treatment. After introducing a *dn-JNK* or treatment with a JNK inhibitor, the percentages of DNA fragmentation in LNCaP cells with or without treatment with PL plus Dox were analysed by a flow cytometer. Error bars represent the s.d. over five independent experiments.

To further determine the nature of the synergistic effect of PL on Dox-induced apoptosis, a DNA fragmentation assay was conducted in the presence of caspase or JNK inhibitors (Figure 5). A general caspase inhibitor z-VADfmk completely blocked the apoptotic process induced by the co-treatment with PL and Dox. Furthermore, the magnitude of apoptosis elicited by the combination treatment was significantly suppressed by the addition of a JNK inhibitor or the introduction of a *dn-JNK* into LNCaP cells, which again supports the notion that PL, via activating JNK and further blocking c-FLIP_L_, potentiates Dox-induced apoptosis.

## DISCUSSION

In the apoptotic process induced by the co-treatment of PL and Dox, caspase 8 is activated, which then initiates a caspase cascade, resulting in the release of cytochrome *c* from the mitochondria to the cytosol. The suppression of caspase activity by a generic caspase inhibitor z-VADfmk completely abolishes apoptosis induced by the co-treatment. Furthermore, the addition of PL alone is able to cause JNK activation, which subsequently downregulates the expression level of c-FLIP_L_ in the cells. The addition of a JNK inhibitor or introduction of a *dn-JNK* suppresses the apoptotic process induced by the co-treatment. Therefore, our findings suggest that PL is a useful synergiser for augmenting Dox-mediated, apoptotic signalling. As such synergy on the induction of apoptosis only requires low doses of Dox, PL has potential for further developing a better therapeutic strategy to treat prostate cancer and avoid the side effects caused by high doses of Dox.

Because prostate cancer progression is partially affected by development of resistance of the cancer cells to apoptosis induced by anticancer drugs, the ability to sensitise apoptotic signalling pathways has important therapeutic implications. Genetic targeting or pharmacological manipulation of caspase family members and their regulators/modulators would provide better clinical strategies. Targeting the expression of caspase 7 has been demonstrated to be a useful approach for treating prostate cancer (Marcelli *et al*, 1998, 1999). In this approach, overexpression of caspase 7 in LNCaP cells could effectively induce apoptosis and has been shown to be very promising clinically (Marcelli *et al*, 1998, 1999). Therefore, the combination treatment of low doses of PL and Dox to activate caspases would have similar implications for treating prostate cancer patients.

Studies using specific caspase inhibitors have shown that Dox can activate caspases 8, 6 and 3 (Hannun, 1997; Los *et al*, 1997). The synergistic effect of Dox and TRAIL on caspase-induced apoptosis has been reported (Wu *et al*, 2002). Here, we demonstrated that Dox is also able to synergise with PL at low doses for the induction of apoptosis. In this apoptotic process, caspases 3, 8 and BID are activated, leading to the release of cytochrome *c* from the mitochondria to the cytosol. A generic caspase inhibitor blocked this synergistic, apoptotic effect on LNCaP cells, further suggesting the involvement of caspase activity in the cytotoxicity rendered by the combination treatment with PL and Dox. However, at low doses neither Dox nor PL elicited the activity of these caspase family members to induce apoptosis. It is possible that treatment with PL at low doses reduces the threshold of the sensitivity of LNCaP cells to Dox-induced apoptosis.

JNK is activated in response to various stresses. The exact mechanisms through which JNK participates in apoptosis remain unknown. JNK is not a direct apoptotic executor, however, instead, it often modulates the initiation of apoptosis through promoting processing of proapoptotic factors, such as the BH3-domain proteins or decaying of antiapoptotic factors (Luo *et al*, 1998). In caspase-induced apoptosis, JNK has been suggested to act at a step upstream to caspase 8 (Deng *et al*, 2003). Recently, experiments, *in vitro* and *in vivo*, have shown that JNK, during TNF*α*-induced apoptotic processes, activates ubiquitin ligase Itch, which specifically interacts with c-FLIP_L_ to promote the degradation of this caspase inhibitor (Chang *et al*, 2006). In our experimental settings, a low dose of PL was enough to elicit JNK phosphorylation, but not to induce apoptosis. However, this activation of JNK was responsible for the reduction of c-FLIP_L_ expression in LNCaP cells. Therefore, JNK, by promoting the degradation process, suppresses c-FLIP_L_, and may sensitise the prostate cancer cells to apoptosis. Doxorubicin is known to be able to elicit cellular or DNA damage-induced responses at low-dose ranges and mobilise caspase family members to execute cell death programs at high-dose ranges. It is well possible that JNK, by lowering the threshold of LNCaP cells to apoptosis, synergises with Dox for the activation of caspases.

*Phellinus linteus* and Dox, at low doses, are G_1_ arrest inducers (Guo *et al*, 2006). The data from colony formation assays demonstrated that LNCaP cells formed fewer colonies in soft agar medium in the presence of a low dose of Dox or PL alone. In contrast, the combination treatment with these two agents at low doses blocks LNCaP cells from growing and forming colonies in soft agar medium. The reduction of the numbers of colonies following treatment with Dox or PL alone at low concentrations is highly likely due to a negative effect of the treatment on cell proliferation, resulting in slow formation of colonies. In addition, the combination treatment of low doses of PL and Dox is nontoxic to normal prostate epithelial cells, causing only growth cessation. This suggests that PL can selectively sensitise cancer cells for apoptotic signalling.

The development of chemotherapeutic resistance to drugs is a major obstacle to the successful treatment of prostate cancer. A more effective anticancer therapy is required. Our present study demonstrates that PL acts as an enhancer to sensitise Dox-mediated, apoptotic signalling, and this sensitisation can be obtained at subtoxic concentrations of Dox. We previously have reported that PL, even at high doses, only causes normal fibroblasts or lung epithelial cells to arrest in the G_1_ phase of the cell cycle (Guo *et al*, 2006). Altogether, our data suggest that PL is an apoptotic synergiser for conventional chemotherapeutics such as Dox, which can keep normal, surrounding cells unharmed. Therefore, PL, serving as a safe alternative medicine, has potential for more efficient therapies against drug-resistant prostate cancer.

## Figures and Tables

**Table 1:** Colony formation of LNCaP cells in soft agar

**Treatment**	**No. of colonies**	**s.d.**
No treatment	174	±7.5
PL (0.5 *μ*g ml^−1^)	87	±5
Dox (1.0 *μ*g ml^−1^)	65	±0.2
Dox (5.0 *μ*g ml^−1^)	0	±0.8
PL (0.5 *μ*g ml^−1^)+Dox (1 *μ*g ml^−1^)	4	±0.5

Dox=doxorubicin; PL=*Phellinus linteus*.

The cells were cultured in soft agar in the presence of PL, Dox, or the combination of PL and Dox for 2 weeks. s.d.: standard deviation over five independent experiments.
